# 

**Published:** 1850-04-01

**Authors:** 


### BOOKS, &c., RECEIVED FOR REVIEW.

The Transactions of the American Medical Association. Vol. II. (A valuable record of important facts.)
A Plea of Humanity in behalf of Medical Education, by A. H. Stevens, M.D. New York, 1849. (An admirable essay.)
Eighteenth Report of the Inspectors of the Eastern State Penitentiary of Pennsylvania.
Fifth and Sixth Reports of the Ohio Lunatic Asylum.
Medical Statistics relating to the Population of Philadelphia, by G. Emerson, M.D.
Code of Ethics of the American Medical Association.
Observations on Delirium Tremens, by S. Jackson, M.D. New York.
Twelfth Annual Report of the Trustees of the State Lunatic Asylum, Worcester. (In our next.)
Mental Pathology, by Dr. Davy. (Will be reviewed in our next.)

We have to express our great obligation to Dr. Isaac Hays, of Philadelphia, for his kindness in forwarding us a body of valuable reports of American Lunatic Asylums, some of recent, and many of early date. We purpose giving an analysis of these documents in a future number. We trust to have it in our power to reciprocate Dr. Hay's civility.

The Reports of the Pennsylvania Hospital for the Insane have been received from Dr. Kirkbridge, the Physician to the Institution. They will be reviewed in our next.
A Letter to the Rev. Thomas Mackreth, Chairman of the Committee of Visitors, on the Detention of Criminal Lunatics in the County Asylum, Lancaster, by the Medical Officers, 1850.
The Pathology of the Kidney in Scarlatina, illustrated by Cases, by J. Miller, M.D. (An excellent work.)
Report of the Littlemore Asylum for 1849.
Tenth Annual Report of the Royal Critchton Institution, Dumfries. (In our next.)
Practical Views on Nervous Diseases, by G. Calvert Holland, M.D. 1850.
Fifth Report of the Hanwell Asylum, 1850. (In our next.)
On Parturition and Obstetrics, by Tyler Smith, M.D. (The psychological portion of this very able work will be noticed in our next number.)
The Chrono-Thermalist, No. I., edited by S. Dickson, M.D.
Report of the Warneford Hospital for the Insane, Headington Hill, near Oxford, for 1849.
Evening Thoughts, by a Physician, 1850.
Somerset County Asylum. Second Report.

---

### JOURNALS RECEIVED IN EXCHANGE.

The London Journal of Medicine.
The Journal of Education.
The Zoist.
The Journal of Prophecy.
The Homœopathic Journal.
The American Journal of Medical Sciences, edited by Dr. Isaac Hays. (We shall notice this specially in our next Number. This is one of the most able of the American Medical Reviews.)
The American Journal of Insanity. (In our next.)
New York Journal of Medicine—regularly.
Dr. Ranking's Half-Yearly Abstract of Medical Sciences.
The London Journal of Arts, Sciences, and Manufactures—regularly.

